# Diseases of the Rectum and Anus

**Published:** 1890-11

**Authors:** 


Diseases of the Rectum and Anus. Their Pathology, Diagnosis and Treatment. By Charles B. Kelsey, A. B., M. D., New York, Professor of Diseases of the Rectum at the New York Post-Graduate Medical School and Hospital; Late Professor of Diseases of the Rectum at the University of Vermont, etc. Third edition Rewritten and Enlarged, with two Chromo-Lithographs and One Hundred and Sixty-eight Illustrations. Wm. Wood & Co., New York.

The third edition of this excellent work, following so closely upon its predecessors, is sufficient evidence of the great esteem in which it is held by the profession.

Many important changes, necessitated by the rapid recent advances in the surgery of the rectum and intestinal surgery generally, have been made; and many valuable additions and illustrations evidence the painstaking care and labor which the author has bestowed upon this edition.

"The chapters on the treatment of stricture, both benign and malignant, and on the formation and closure of artificia anus, have been entirely rewritten."

The author, while he differs with some of the best rectal specialists in several particulars, explains fully his reasons for those differences.

The large clinical experience of the writer and his thorough familiarity, from a wide experience as a teacher, with the needs of the general practitioner, together with his clear, forcible style, have combined to make the work eminently practical.

